# Acute Polyradiculomyelitis With Spinal Cord Gray Matter Lesions: A Report of Two Cases

**DOI:** 10.3389/fneur.2021.721669

**Published:** 2021-08-19

**Authors:** Charidimos Tsagkas, Maria Janina Wendebourg, Matthias Mehling, Johannes Lorscheider, Philippe Lyrer, Bernhard Friedrich Décard

**Affiliations:** Neurology Clinic and Policlinic, Departments of Medicine, Clinical Research and Biomedical Engineering, University Hospital Basel and University of Basel, Basel, Switzerland

**Keywords:** MRI, peripheral neuropathology, myelopathy, guillain-barre syndrome, clinical neurology, spinal cord gray matter lesions, AIDP

## Abstract

**Objective:** Inflammatory polyradiculomyelitis belongs to a rare group of immune-mediated diseases affecting both the central and peripheral nervous system. We aimed to describe an unusual presentation of acute polyradiculomyelitis with marked spinal cord lesions restricted to the gray matter.

**Methods:** Thorough examination of two case reports including clinical, MRI, serologic, electrophysiologic and CSF examinations as well as short-term follow-up.

**Results:** We present two adult patients with acute polyradiculomyelitis and unusual spinal cord lesions restricted to the gray matter on MRI. The clinical presentation, serologic, electrophysiologic and CSF features of the two patients varied, whereas both patients demonstrated severe, asymmetrical, predominantly distal, motor deficits of the lower extremities as well as bladder and bowel dysfunction. Both patients only partially responded to anti-inflammatory treatment. Severe motor impairment and bladder dysfunction persisted even months after symptom onset.

**Conclusions:** To our best of knowledge, these are the first reports of acute polyradiculomyelitis with distinct involvement of the lower thoracic spinal cord gray matter. Currently, it remains unclear whether gray matter lesions reflect a separate pathophysiologic mechanism or an exceedingly rare presentation of spinal cord involvement in acute polyradiculomyelitis.

## Introduction

Inflammatory polyneuroradiculopathies are a group of immune-mediated diseases of the peripheral nerves and their spinal roots (1). Clinical manifestation usually involves distal onset and ascending sensory and/or motor deficits as well as autonomic dysfunction. Often, symptoms follow a preceding infection, most commonly with C. jejuni or respiratory viral pathogens (2). Symptom onset and progression may be acute or slow over a longer time span. Typical electrophysiological findings include slowing of nerve conduction velocity (NCV), conduction blocks and F-wave alterations, although early in the disease process, changes may not be detected (3). CSF analysis typically shows albuminocytological dissociation. The most effective therapy is intravenous immunoglobulin (IVIG) whereas rapid administration, especially in acute cases, is important.

Most common MRI findings include thickening of the cauda equina and spinal roots (4). Contrast enhancement of the conus, cauda equina and spinal roots is also compatible with the diagnosis (5), whereas it may disappear after treatment (6).

Rarely, myelitis accompanies inflammatory polyneuroradiculitis (7), resulting in polyradiculomyelitis. Viral or bacterial pathogens may be detected (8, 9).

However, to our knowledge, the combination of inflammatory polyneuroradiculitis and spinal cord (SC) lesions restricted to the gray matter (GM) has not been reported before. We present two cases of acute-onset inflammatory polyradiculomyelitis with GM lesions.

## Case Reports

### First Case

A 56-year-old male with no previous neurologic history presented with severe lower back pain and reduced sensation as well as weakness of the distal right lower extremity. Within the next hours, these symptoms extended to the left leg and ascended proximally. Additionally, he complained about urinary retention and obstipation.

Clinically, he showed a predominantly right-sided, severe, flaccid, distal paraparesis and hypoesthesia involving dermatomes L4-S5 in all sensory modalities of both lower extremities. Spontaneous fasciculations were visible on the right M. quadriceps femoris. Stretch reflexes were absent in both lower extremities except for normal left quadriceps and adductor reflexes.

**Figure 1:**
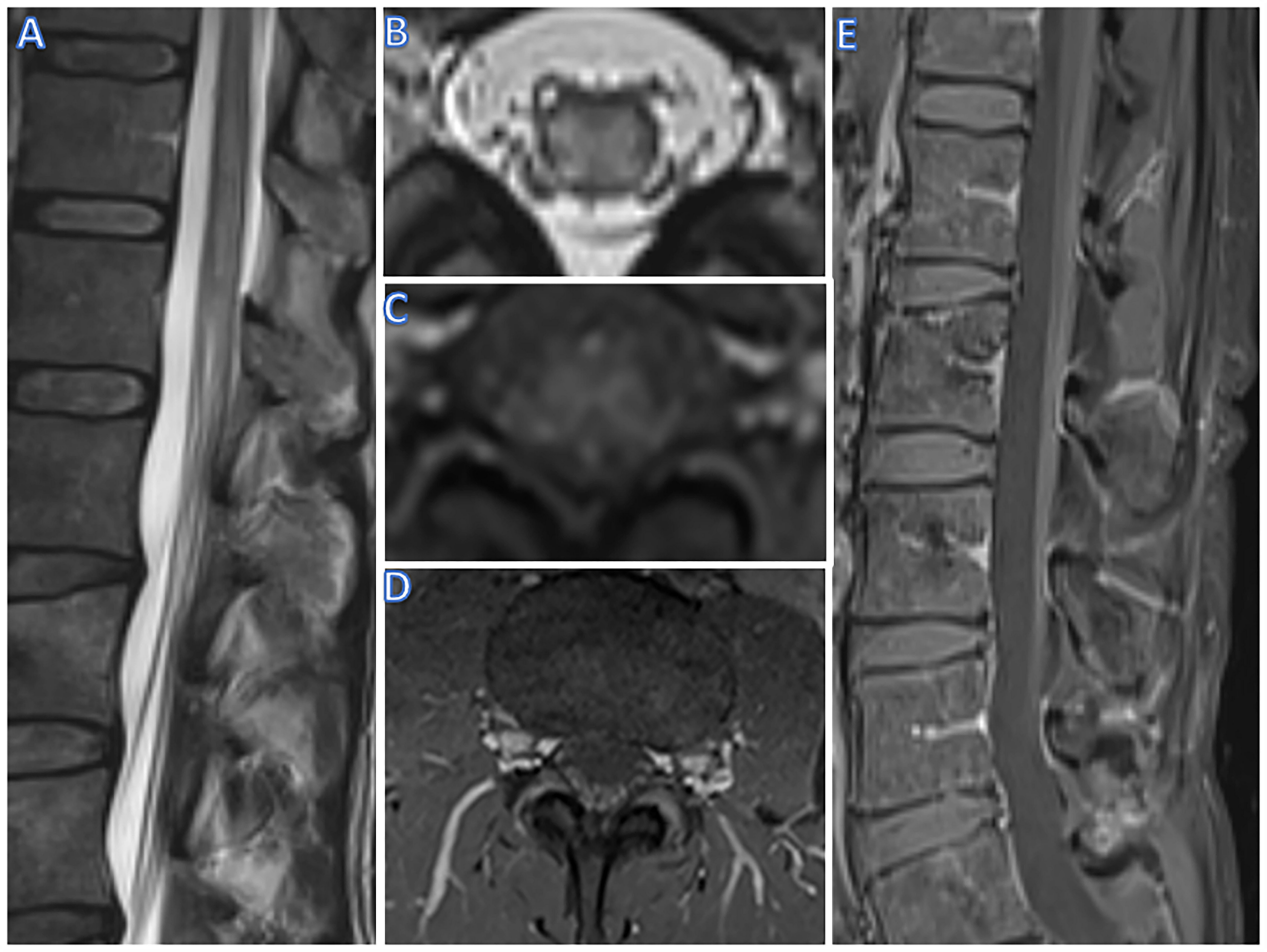
R imaging of case 1 (male, 56 yrs). T2w hyperintense lesions and swelling of the conus **(A)** and selective hyperintensity and swelling of the gray matter on axial imaging **(B)**. On T1w, a diffuse contrast enhancement of the gray matter can be seen **(C)**, as well as contrast enhancement of spinal roots in axial **(D)** and sagittal imaging **(E)**.

SC T2-weighted MRI of the lumbar spine revealed a hyperintense lesion extending from T12 to L1, including the conus medullaris with a butterfly-like shape on axial slices 1 day after admission. Post-contrast T1-weighted MRI showed enhancement of the cauda equina and subtle contrast enhancement of the SC lesion. A follow-up MRI 3 days later showed focal SC edema and persistence of subtle lesion contrast enhancement (Figures 1A–E). CSF analysis revealed an albuminocytological dissociation as well as an increased CSF/serum albumin quotient (Table 1). Serological analysis was positive for Campylobacter jejuni. Anti-ganglioside antibodies were unremarkable. NCV studies showed increased distal motor latencies. F-waves were either prolonged or absent (Table 1).

**Table 1:** Overview of diagnostic measures applied to both patients.

	**Patient A (male, 55 yrs)**	**Patient B (female, 62 yrs)**
Serum analysis (pathological findings)	- Campylobacter jejuni titer: 1:96	- not measured
Further serum analysis (unremarkable findings)	- Anti-aquaporin-4 Ab: not measured - Anti-MOG Ab: not measured - Anti-ganglioside Ab negative - HIV screen negative - Lues screen negative - EBV, VZV, HSV I/II, CMV IgM negative - Poliomyelitis neutralization test: not measured	- Anti-aquaporin-4 Ab: negative - Anti-MOG Ab: negative - Anti-ganglioside Ab: negative - HIV screen: negative - Lues screen: negative - EBV, VZV, HSV I/II, CMV IgM:negative - Poliomyelitis neutralization test:proven immunity
CSF analysis	- Total protein 1,292 mg/l - Leukocytes 0 × 10^6^/l - CSF/serum albumin quotient 20.1 × 10^−3^	- Total protein 395 mg/l - Leukocytes 0 × 10^6^/l - CSF/serumalbumin quotient 5.6 × 10^−3^
Neurography studies	- Day 1 after symptom onset: F-waves of lower extremities absent or elongated; distal motor latencies elongated - Follow-up examination: not conducted	- Day 2 after symptom onset:F-waves of lower extremities absent, CMAP of right EDB reduced - Day 20 after symptom onset:Motor nerves of lower extremities notmeasurable, sensory nerves of lowerextremities intact
Myography studies	Not conducted	Signs of acute denervation in leftgastrocnemius, right TA andgastrocnemius without any sign ofvoluntary or spontaneous activity
MR imaging (brain)	Unremarkable	Unremarkable
MR imaging (spinal cord)	- Day 1 after symptom onset: Gray matter myelopathy from Th11 to conus (L1); slight contrast enhancement of lumbar radices - Day 3 after symptom onset: Stationary gray matter myelopathy from Th11 to conus (L1); stationary slight contrast enhancement of lumbar radices	- At symptom onset:Gray matter myelopathy at Th11/12, no contrast enhancement at this point - Day 22 after symptom onset:Gray matter myelopathy at Th11/12; now contrast enhancement of lumbar radices visible

*Ab, antibodies; CMAP, compound muscle action potential; CMV, cytomegalovirus; CSF, cerebrospinal fluid; EBV, Epstein-Barr virus; EDB, M. extensor digitorum brevis; HSV I/II, herpes simplex virus type 1 & 2; MOG, myelin oligodendrocyte glycoprotein; TA, tibialis anterior; VZV, varicella-zoster virus*.

Hence, acute post-infectious polyradiculomyelitis was diagnosed. The patient was treated with IVIG 35g/d for 5 days. Under IVIG treatment, the patient improved significantly, particularly regarding proximal motor deficits, but was dismissed with a persisting severe, predominantly right-sided and distal, flaccid paraparesis and hypoesthesia as well as urinary retention and obstipation. The patient was transferred to a neuro-rehabilitation facility. After 3 months, his symptoms improved slightly further, but paraparesis and bladder dysfunction remained.

### Second Case

A 62-year-old female with no previous neurologic history presented with acute pain and weakness in both lower extremities.

Clinically, she showed a mild, predominantly right-sided and distal paraparesis. The anal sphincter tonus was normal at that time point. Within a few hours, her symptoms deteriorated dramatically, and she developed a severe, flaccid, predominantly right-sided, distal paraparesis accompanied by urinary and stool incontinence. The patellar reflex was absent on the right side and brisk on the left side, whereas the achilles tendon reflexes were absent bilaterally.

**Figure 2:**
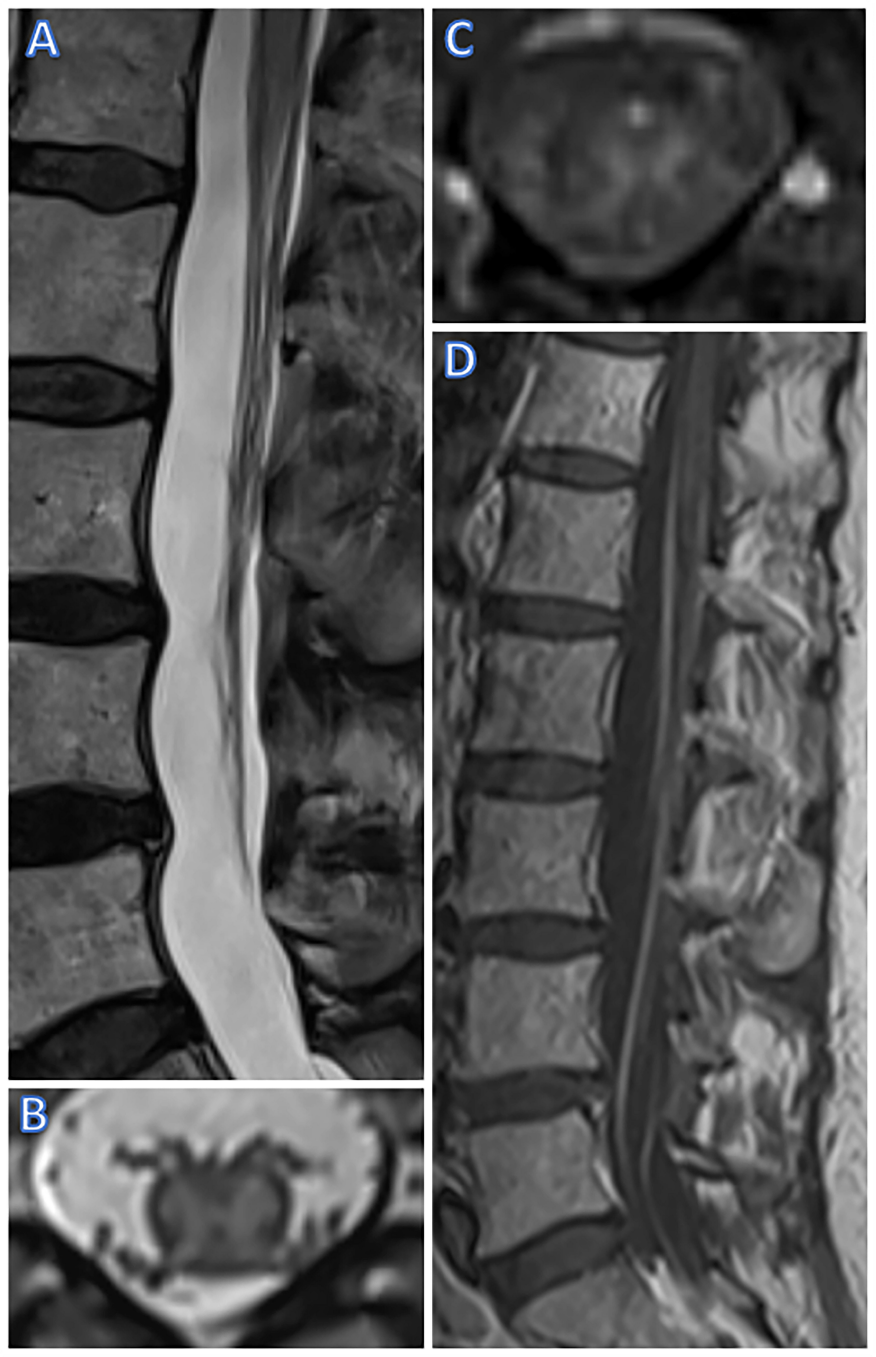
MR Imaging of case 2 (female, 63 yrs). T2w sagittal imaging shows swelling and hyperintense lesions of the conus **(A)** while axial T2w imaging shows a butterfly-shaped lesion involving only the gray matter [at T11-12, **(B)**]. T1w imaging showed discreet contrast enhancement of the anterior horns on axial imaging **(C)**, and, on day 22 after onset, post-contrast T1w imaging also showed contrast of spinal cord roots **(D)**.

SC T2-weighted MRI revealed a hyperintense lesion from T11 to L1, including the conus medullaris, with a butterfly-like shape on axial slices. Post-contrast T1-weighted MRI showed partial enhancement of the anterior parts of this lesion (Figures 2A–D). Brain MRI and CSF-analysis were unremarkable (Table 1). Two days after symptom onset, NCV studies showed absent F-waves of both lower extremities and a reduced compound muscle action potential of the M. extensor digitorum brevis. Electromyography demonstrated neither spontaneous nor voluntary activity of the right M. gastrocnemius, whereas examination of the left M. tibialis anterior showed signs of acute denervation. Somatosensory evoked potentials of tibial nerve were unremarkable on both sides. Motor evoked potentials of the right lower extremity were absent, whereas normal latencies were shown in the left lower extremity. Serologic analysis was unremarkable (Table 1). A poliovirus neutralization-test showed immunity.

Therefore, idiopathic acute polyradiculomyelitis was diagnosed. Assuming the myelitis was the leading cause for the symptoms, the patient was initially treated with methylprednisolone i.v., 500 mg daily for 5 days and consecutive oral prednisone. Under this regimen, the patient's symptoms improved slightly, but the paraparesis, bladder and bowel dysfunction were still severe. Therefore, 6 cycles of plasmapheresis were carried out. A further slight improvement of the motor symptoms became evident.

Thereafter, electrophysiological studies were repeated. Distal motor potentials of tibial and peroneal nerves were absent, whereas proximally (left N. femoralis), a compound muscle action potential amplitude reduction was shown. In contrast, sensory NCV studies of the lower extremities, e.g., the left sural and superficial peroneal nerve, were normal. Furthermore, the upper extremities showed normal NCV studies. Electromyography of the right vastus lateralis muscle showed spontaneous activity and increased recruitment frequency (>20/s). In addition, a follow-up SC MRI (22 days after the first MRI) showed new post-contrast enhancement of the cauda equina inT1-weighted MRI (Figure 2D). The SC T2-weighted hyperintensity remained stationary and showed subtle post-contrast enhancement in T1-weighted imaging.

After 3 months of neurorehabilitation, proximal pareses of the lower extremities were slightly improved but distal motor and bladder dysfunction persisted.

## Discussion

We present two cases of acute polyradiculomyelitis with distinct involvement of the lower SCGM on MRI. Lesions restricted to the SCGM are not a common feature of acute polyradiculomyelitis. They have been described in acute SC infarction or chronic compressive myelopathy, leading to the typical “snake/owl's eyes” or “fried eggs” appearance on axial T2-weighted images (10, 11). In contrast to our patients, contrast enhancement in SC ischemia is absent in the acute phase. In addition, anterior horn lesions have been reported in some rare cases of West Nile virus-associated myelitis (12). However, due to a lack of a relevant travel history and missing typical accompanying symptoms such as high fever, neither patient was tested for West Nile virus. In addition, both patients formally fulfill the diagnostic criteria of the herpes simplex virus-associated Elsberg syndrome as described by Savoldi and colleagues (13) with the first patient meeting the criteria for a clinically definite and the second patient for a clinically probable diagnosis. However, in contrast to patients with Elsberg syndrome, our patients did not report signs of previous herpes simplex virus infection. Furthermore, Gorson and Ropper described two myelitis cases with polio-like anterior horn lesions mimicking a motor Guillain-Barre syndrome variant after an unspecific mild viral infection (14). Neither of our patients had reported symptoms of a previous viral infection. However, mild symptoms may simply not have been acknowledged. Moreover, polyradiculomyelitis has also been described in the context of both aquaporin-4 antibody positive neuromyelitis optica spectrum disorder and myelin oligodendrocyte glycoprotein (MOG) associated disorder (15–18). In particular, MOG-associated disorder frequently presents with longitudinally extensive lesions restricted in the SCGM of the thoracolumbar region, typically with absent contrast enhancement; in contrast, in patients with aquaporin-4 antibody positive myelitis, cervical and thoracic longitudinally extensive lesions with contrast enhancement are more frequent (19, 20). Indeed, both these differential diagnoses were considered in the clinical management of one of our patients (patient 2), which —however— tested negative for both anti-aquaporin 4 and anti-MOG antibodies. In addition, patient 1, who was not tested for any of those antibodies, did not demonstrate any additional typical signs and features of these disorders such as longitudinally extensive spinal cord lesions, brain or optic nerve involvement, recurrent and/or multifocal disease making these etiologies rather unlikely.

The underlying pathology for these MRI findings is unclear. Possibly, they reflect the aftermath of axonal injury occurring distally to the SCGM and leading to secondary Wallerian degeneration and axonal swelling reaching the neuron-somas. However, this hypothesis does not explain the presence of these lesions at symptom onset in our patients since axonal degeneration begins 36–44 h after nerve injury. Hence, a direct SCGM inflammatory involvement seems more plausible, which is also supported by the SCGM contrast enhancement in one of our patients. Despite the SC involvement in both patients, no pleocytosis was shown in CSF analysis, whereas one patient showed typical albuminocytological dissociation. Regarding the pathogenesis of our patients' disorder, bacterial and viral agents such as Campylobacter jejuni, Mycoplasma pneumoniae as well as the Zika and Dengue viruses have been described as triggers in both transverse myelitis and inflammatory polyneuroradiculitis (21). One of our patients tested positive for Campylobacter jejuni (the second patient was not tested), which may offer a pathogenetic explanation. Despite that, both patients had negative anti-ganglioside antibodies, which have been associated with these pathogens, although this is not unusual in all forms of polyneuroradiculitis (21).

With regard to the clinical features, both patients presented with atypical polyneuroradiculitis symptoms e.g., acute, distal, flaccid paralysis of the lower extremities, but also marked bowel and bladder symptoms. The latter most likely correspond to the SC involvement rather than the polyneuropathic component of the patients' disorder.

Notably, besides SC-associated symptoms, the two patients presented with different phenotypes; while the first patient showed sensorimotor deficits, the second patient presented a pure motor-fiber involvement. In patients with Elsberg syndrome, sensory nerve involvement may not be present. The pure motor fiber involvement may reflect the pronounced anterior horn lesions. Finally, despite early immunomodulatory treatment, severe motor impairment and bladder dysfunction persisted even months after symptom onset. In the few reported cases of concomitant myelopolyradiculitis (22–24), long-term clinical outcomes varied, although pure motor variants seemed to be associated with a poorer prognosis (25).

To summarize, we report two cases of unusual SCGM lesions in patients with acute polyradiculomyelitis. Currently, it remains unclear whether this presentation reflects a separate pathophysiologic mechanism or an underappreciated manifestation of the inflammatory disease. Hence, future larger-scale studies should further investigate these findings.

## Data Availability Statement

The original contributions presented in the study are included in the article/supplementary material, further inquiries can be directed to the corresponding authors.

## Ethics Statement

Ethical review and approval was not required for the study on human participants in accordance with the local legislation and institutional requirements. The patients/participants provided their written informed consent to participate in this study. Written informed consent was obtained from the individuals for the publication of any potentially identifiable images or data included in this article.

## Author Contributions

CT, MW, and BD drafted the manuscript and reviewed the data reported. All authors contributed to the clinical management of the reported patients and the revision and editing of the manuscript.

## Conflict of Interest

The authors declare that the research was conducted in the absence of any commercial or financial relationships that could be construed as a potential conflict of interest.

## Publisher's Note

All claims expressed in this article are solely those of the authors and do not necessarily represent those of their affiliated organizations, or those of the publisher, the editors and the reviewers. Any product that may be evaluated in this article, or claim that may be made by its manufacturer, is not guaranteed or endorsed by the publisher.

## Abbreviations

NCV: nerve conduction velocity
IVIG: intravenous immunoglobulin
MOG: myelin oligodendrocyte glycoprotein
SC: spinal cord
GM: gray matter.

## References

[1] Nobile-Orazio E. Chronic inflammatory demyelinating polyradiculoneuropathy and variants: where we are and where we should go. J Peripher Nerv Syst JPNS. (2014) 19:2–13. 10.1111/jns5.12053 24612201

[2] Hadden RD Karch H Hartung HP Zielasek J Weissbrich B Schubert J. Preceding infections, immune factors, and outcome in Guillain-Barré syndrome. Neurology. (2001) 56:758–65. 10.1212/WNL.56.6.758 11274311

[3] Berciano J Orizaola P Gallardo E Pelayo-Negro AL Sánchez-Juan P Infante J. Very early Guillain-Barré syndrome: a clinical-electrophysiological and ultrasonographic study. Clin Neurophysiol Pract. (2020) 5:1–9. 10.1016/j.cnp.2019.11.003 31886449 PMC6923288

[4] Byun WM Park WK Park BH Ahn SH Hwang MS Chang JC. Guillain-Barré syndrome: MR imaging findings of the spine in eight patients. Radiology. (1998) 208:137–41. 10.1148/radiology.208.1.9646804 9646804

[5] Alkan O Yildirim T Tokmak N Tan M. Spinal MRI findings of Guillain-Barré syndrome. J Radiol Case Rep. (2009) 3:25–8. 10.3941/jrcr.v3i3.153 22470650 PMC3303301

[6] Iwata F Utsumi Y. MR imaging in Guillain-Barré syndrome. Pediatr Radiol. (1997) 27:36–8. 10.1007/s002470050059 8995165

[7] Guo F Zhang Y-B. Clinical features and prognosis of patients with Guillain-Barré and acute transverse myelitis overlap syndrome. Clin Neurol Neurosurg. (2019) 181:127–32. 10.1016/j.clineuro.2019.04.014 31039494

[8] Poorthuis MHF Battjes S Dorigo-Zetsma JW de Kruijk JR. Primary Epstein-Barr virus infection in immunocompetent patients with acute transverse myelitis and a combination of polyradiculitis and anterior horn syndrome as neurological manifestations. BMJ Case Rep. (2018) 2018:bcr2018225333. 10.1136/bcr-2018-225333 30158264 PMC6119397

[9] Canpolat M Kumandas S Yikilmaz A Gumus H Koseoglu E Poyrazoglu HG. Transverse myelitis and acute motor sensory axonal neuropathy due to legionella pneumophila: a case report. Pediatr Int Off J Jpn Pediatr Soc. (2013) 55:778–82. 10.1111/ped.12126 24330286

[10] Al-Mefty O Harkey LH Middleton TH Smith RR Fox JL. Myelopathic cervical spondylotic lesions demonstrated by magnetic resonance imaging. J Neurosurg. (1988) 68:217–22. 10.3171/jns.1988.68.2.0217 3339437

[11] Novy J Carruzzo A Maeder P Bogousslavsky J. Spinal cord ischemia: clinical and imaging patterns, pathogenesis, and outcomes in 27 patients. Arch Neurol. (2006) 63:1113–20. 10.1001/archneur.63.8.1113 16908737

[12] Kalita J Vibhute A Kumar M Misra UK. Myelopathy in west Nile virus encephalitis: report of a case and review of literature. J Spinal Cord Med. (2020) 43:444–8. 10.1080/10790268.2018.1507804 30124385 PMC7480635

[13] Savoldi F Kaufmann TJ Flanagan EP Toledano M Weinshenker BG. Elsberg syndrome: a rarely recognized cause of cauda equina syndrome and lower thoracic myelitis. Neurol Neuroimmunol Neuroinflammation. (2017) 4:e355. 10.1212/NXI.0000000000000355 28534040 PMC5427668

[14] Gorson KC Ropper AH. Nonpoliovirus poliomyelitis simulating Guillain-Barré syndrome. Arch Neurol. (2001) 58:1460. 10.1001/archneur.58.9.1460 11559319

[15] Rinaldi S Davies A Fehmi J Beadnall HN Wang J Hardy TA. Overlapping central and peripheral nervous system syndromes in MOG antibody–associated disorders. Neurol Neuroimmunol Neuroinflammation. (2020) 8:e924. 10.1212/NXI.0000000000000924 33272955 PMC7803332

[16] Takai Y Misu T Nakashima I Takahashi T Itoyama Y Fujihara K. Two cases of lumbosacral myeloradiculitis with anti-aquaporin-4 antibody. Neurology. (2012) 79:1826–1828. 10.1212/WNL.0b013e3182703ff7 23054238

[17] Kim S Park J Kwon BS Park J-W Lee HJ Choi J-H. Radiculopathy in neuromyelitis optica. How does anti-AQP4 Ab involve PNS? Mult Scler Relat Disord. (2017) 18:77–81. 10.1016/j.msard.2017.09.006 29141825

[18] Toru S Soejima I Katayama Y Saito K Yokote H. A case of anti-AQP4 antibody-positive neuromyelitis optica spectrum disorder with MRI-proven lesions in lumbar nerve roots. Mult Scler Relat Disord. (2020) 46:102557. 10.1016/j.msard.2020.102557 33296967

[19] Dubey D Pittock SJ Krecke KN Morris PP Sechi E Zalewski NL. Clinical, radiologic, and prognostic features of myelitis associated with myelin oligodendrocyte glycoprotein autoantibody. JAMA Neurol. (2019) 76:301–309. 10.1001/jamaneurol.2018.4053 30575890 PMC6440233

[20] Sato DK Callegaro D Lana-Peixoto MA Waters PJ Jorge de FMH Takahashi T. Distinction between MOG antibody-positive and AQP4 antibody-positive NMO spectrum disorders. Neurology. (2014) 82:474–81. 10.1212/WNL.0000000000000101 24415568 PMC3937859

[21] Tripp A. Acute transverse myelitis and Guillain–Barre overlap syndrome following influenza infection. CNS Spectr. (2008) 13:744–7. 10.1017/S1092852900013845 18849892

[22] Sinha S Prasad KN Jain D Pandey CM Jha S Pradhan S. Preceding infections and anti-ganglioside antibodies in patients with Guillain–Barré syndrome: a single centre prospective case-control study. Clin Microbiol Infect. (2007) 13:334–7. 10.1111/j.1469-0691.2006.01636.x 17391394

[23] Tolunay O Çelik T Çelik Ü Kömür M Tanyeli Z Sönmezler A. Concurrency of Guillain-Barre syndrome and acute transverse myelitis: a case report and review of literature. Korean J Pediatr. (2016) 59:S161–4. 10.3345/kjp.2016.59.11.S161 28018472 PMC5177703

[24] Howell KB Wanigasinghe J Leventer RJ Ryan MM. Concomitant transverse myelitis and acute motor axonal neuropathy in an adolescent. Pediatr Neurol. (2007) 37:378–81. 10.1016/j.pediatrneurol.2007.05.020 17950429

[25] Rodríguez Y Rojas M Pacheco Y Acosta-Ampudia Y Ramírez-Santana C Monsalve DM. Guillain–Barré syndrome, transverse myelitis and infectious diseases. Cell Mol Immunol. (2018) 15:547–62. 10.1038/cmi.2017.142 29375121 PMC6079071

